# Mutation of a common amino acid in NKX2.5 results in dilated cardiomyopathy in two large families

**DOI:** 10.1186/s12881-016-0347-6

**Published:** 2016-11-17

**Authors:** Alan Hanley, Katie A. Walsh, Caroline Joyce, Michael A. McLellan, Sebastian Clauss, Amaya Hagen, Marisa A. Shea, Nathan R. Tucker, Honghuang Lin, Gerard J. Fahy, Patrick T. Ellinor

**Affiliations:** 1 Cardiovascular Research Center, Massachusetts General Hospital, Boston, MA USA; 2 Cork University Hospital, Cork, Wilton Ireland; 3 Program in Medical and Populations Genetics, Broad Institute, Cambridge, MA USA; 4 Department of Medicine, Boston University School of Medicine, Boston, MA USA

**Keywords:** Sudden cardiac death, Cardiomyopathy, Arrhythmia, Next generation sequencing

## Abstract

**Background:**

The genetic basis for dilated cardiomyopathy (DCM) can be difficult to determine, particularly in familial cases with complex phenotypes. Next generation sequencing may be useful in the management of such cases.

**Methods:**

We report two large families with pleiotropic inherited cardiomyopathy. In addition to DCM, the phenotypes included atrial and ventricular septal defects, cardiac arrhythmia and sudden death. Probands underwent whole exome sequencing to identify potentially causative variants.

**Results:**

Each whole exome sequence yielded over 18,000 variants. We identified distinct mutations affecting a common amino acid in *NKX2.5*. Segregation analysis of the families support the pathogenic role of these variants.

**Conclusion:**

Our study emphasizes the utility of next generation sequencing in identifying causative mutations in complex inherited cardiac disease. We also report a novel pathogenic NKX2.5 mutation.

## Background

Dilated cardiomyopathy (DCM) is a condition associated with significant morbidity and mortality that may have identifiable causes or may be idiopathic or inherited. A spectrum of genetic loci associated with DCM has been identified, with over 30 genes implicated [1].

We sought to identify the causative mutation in two unrelated individuals with DCM. Each reported an extensive family history of congenital heart disease (CHD) with multiple cardiac diagnoses affecting several family members. In addition to identifying potential causative mutations, we also sought to address the possibility of a founder effect as both families lived in the same rural geographic area.

To accomplish these goals, we took advantage of the increasing availability and rapidly decreasing cost of next generation sequencing to identify variations within the protein coding region of the genome. We describe the identification of variants potentially implicated in the cardiac disease affecting both families. We also provide a description of our approach to prioritizing variants for further segregation analysis, and the subsequent discovery of a novel *NKX2-5* variant.

## Case presentations

**Fig. 1:**
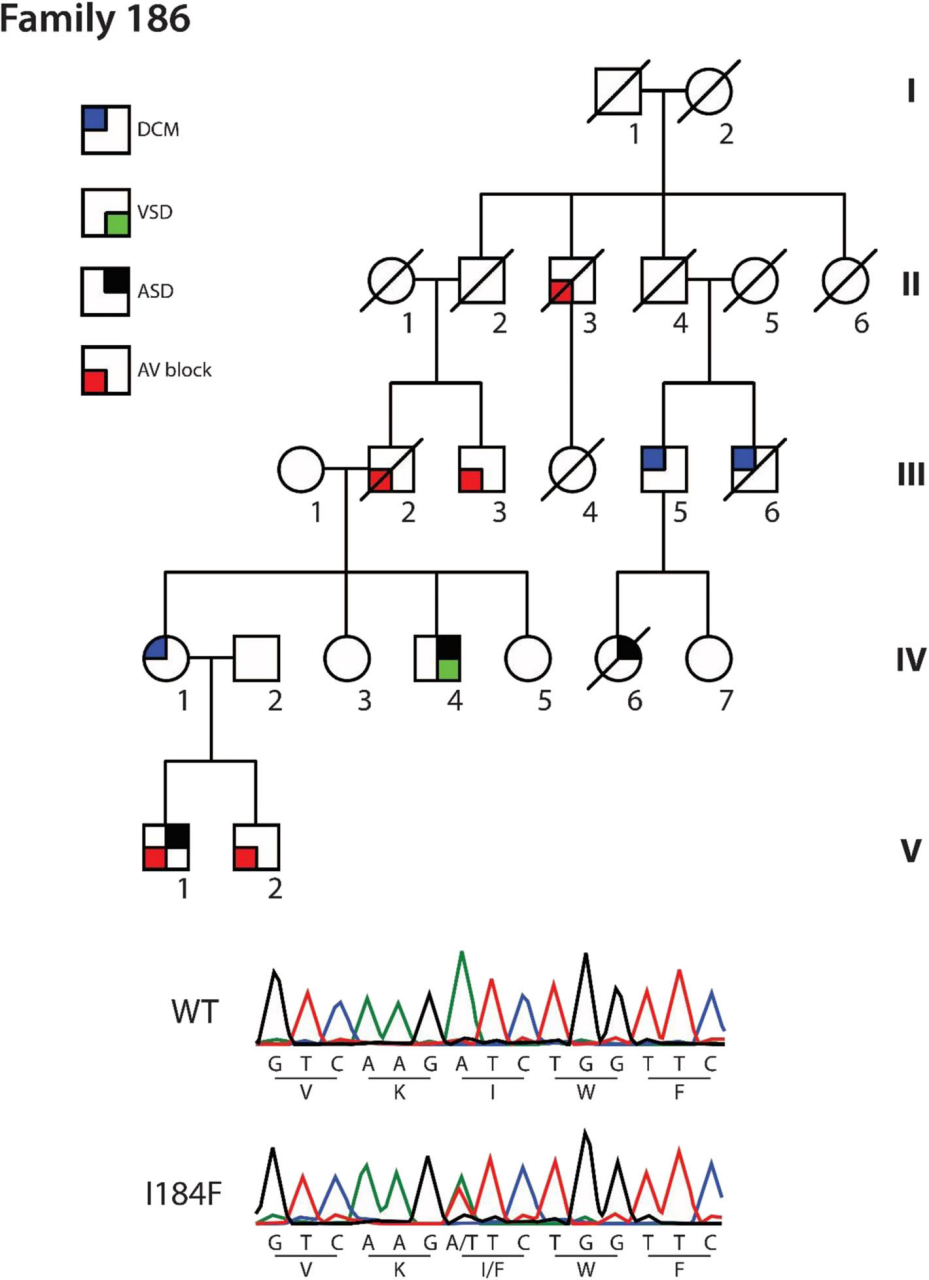
Pedigree showing affected and unaffected individuals in family 186. Alignment chromatograms showing representative wildtype (WT, above) and heterozygous mutant (I184F, below) tracings

A female in her early forties (family 186) presented acutely to hospital with pulmonary edema, atrial fibrillation and non-sustained ventricular tachycardia (VT). She had no prior history of cardiac disease. Echocardiography demonstrated severely impaired left ventricular function. Her son and father had both undergone pacemaker implantation at the age of fifteen and in the fourth decade of life respectively. In addition, there was a history of cardiac disease in the extended family including DCM, atrial and ventricular septal defects and sudden death (Fig 1, Table 1).

**Table 1:** Clinical characteristics of family 186

Pedigree	Genotype	Clinical data
V1	+	CHB, PPM, ASD
V2	+	1st degree AVB
IV.4	+	AVSD, AF
IV.3	+	Normal
IV.5	+	Normal
III.2	NA	PPM
III.3	NA	PPM
II.3	NA	PPM
III.4	NA	SCD
II.6	NA	SCD
III.5	NA	DCM, VT
IV.6	NA	ASD, SCD
III.6	NA	DCM, AF, SCD

**Fig. 2:**
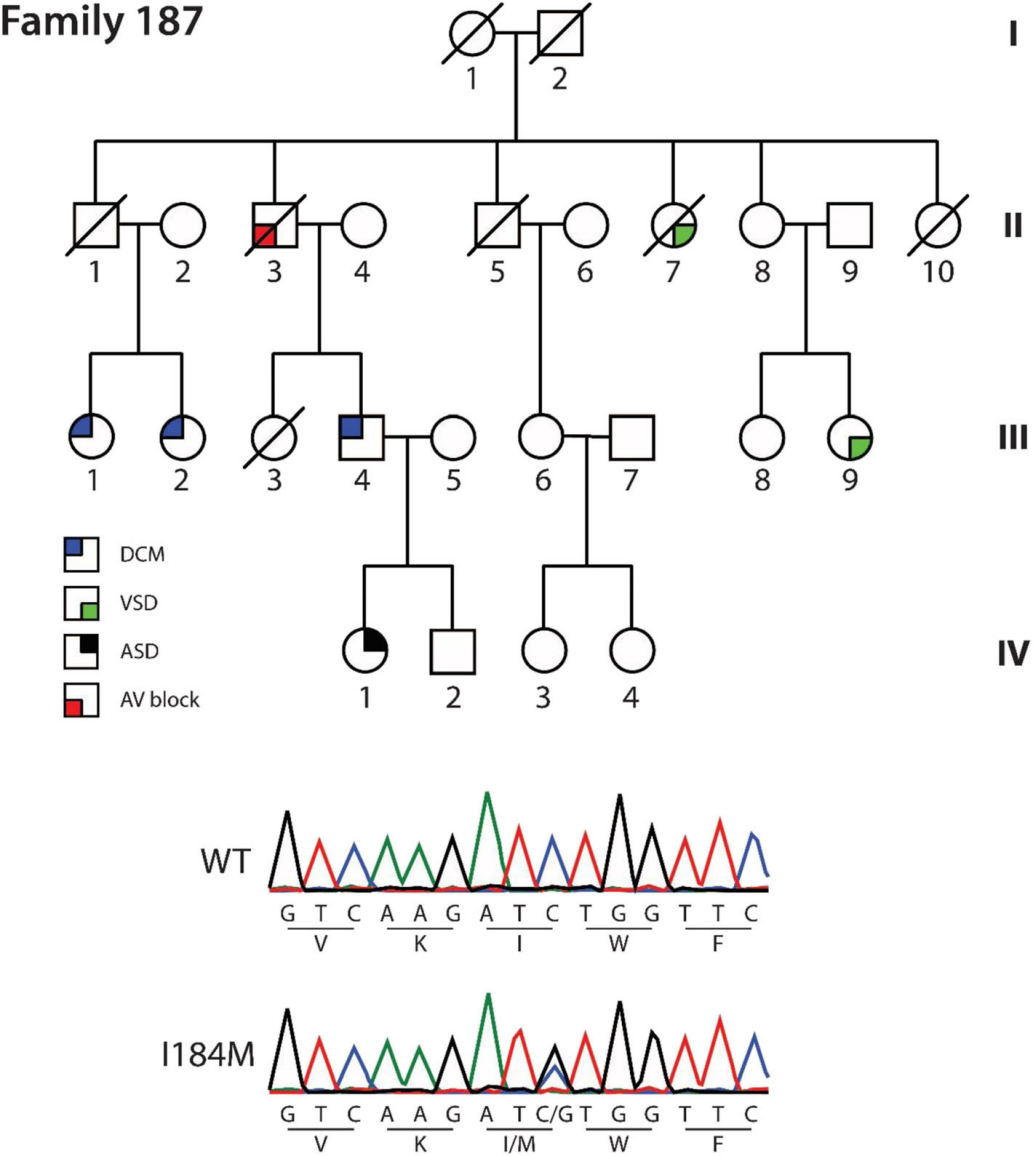
Pedigree showing affected and unaffected individuals in family 187. Alignment chromatograms showing representative wildtype (WT, above) and heterozygous mutant (I184M, below) tracings

A second unrelated proband (family 187) presented to the same practice in her teenage years with DCM and VT treated with an implantable cardioverter-defibrillator. She also had severely impaired left ventricular function as demonstrated on echocardiography. Family history was remarkable for cardiac disease including DCM, atrial and ventricular septal defects and sudden death (Fig 2, Table 2).

**Table 2:** Clinical characteristics of family 187

Pedigree	Genotype	Clinical data
III.2	+	DCM
III.4	+	DCM, heart transplant
IV.1	+	AVSD
III.6	-	VT
III.9	-	VSD
II.1	NA	PPM/ICD
II.3	NA	PPM, SCD
II.5	NA	SCD, CAD
II.7	NA	Tricuspid atresia, right ventricular hypoplasia, PS, VSD
II.10	NA	Cyanotic heart disease, SCD
III.3	NA	SCD

Both families lived in adjacent rural towns with populations of approximately 11,600 and 1,650 people, respectively. Due to the similarities in phenotypes and small referral population we suspected a founder effect may be present, with a common mutation accounting for the observed illnesses. The families were unable to identify a common ancestor, despite well-documented pedigrees extending to the mid-nineteenth century.

### Ethics approval and consent to participate

All participants were enrolled in the ongoing genetics of cardiovascular disease study at Massachusetts General Hospital (MGH). The study was approved by the Institutional Review Board and Human Research Committee at MGH and complied with the Declaration of Helsinki. Written informed consent, including consent to publish was obtained prior to performing the evaluations. The probands agreed to genetic testing by way of next generation sequencing in an effort to identify a causative mutation. Samples were processed with the Roche Large Volume DNA isolation kit using magnetic bead technology, following which genomic DNA was extracted on the Roche MagnaPure Automated DNA extractor.

### Methods

Exome capture was performed using Agilent SureSelect assay (v4) according to the manufacturer’s recommendation. The library was then amplified and pair-end sequenced by the Illumina HiSeq 2500 platform at the Broad Institute (Cambridge, MA). The BWA software package (Version: 0.5.8) was used to map the sequenced reads to the human reference genome (NCBI Build 37, hg19) [2]. The resulted SAM files were then converted into BAM files by samtools (Version: 0.1.18) [3]. The MarkDuplicates function of the Picard software package was used to remove duplicate reads, which were defined as those with the exact same start and end positions. The GATK software package (Version 1.2–21) was used to recalibrate base qualities and perform local realignment around indels. The variant calling was implemented by the UnifiedGenotyper function within GATK software package [4].

Sequencing identified in excess of 18,000 variants in each exome. Filtration and prioritization of the variants was performed according to previously published methods [5]. First, all common variants (>1%) identified in publically available databases (dbSNP [6], 1000 Genomes Project [7],and Exome Variant Server [8]) were removed from further analyses. Second, synonymous variants were excluded on the basis that these would have no effect on protein function. Following this, analysis of evolutionary conservation of the altered amino acids was performed using the Phylop tool. PolyPhen-2, and SNAP were used to predict the effect of the amino acid substitution on protein structure. Variants affecting amino acids that are highly conserved through evolution and those predicted to have a significant effect on protein structure were then initially analyzed based on their known association with human disease.

## Results

An initial review of the observed variants revealed distinct missense heterozygous mutations in *NKX2.5* affecting the same residue in both families. These variants were chosen for further study due to the known role that mutations in *NKX2.5* play in inherited cardiovascular disease in humans. None of the other variants had such a clear, previously identified association with the observed phenotypes.

The residue, Isoleucine184, is found in the important homeodomain region and is highly conserved. The variant affecting family 186 was novel, affecting an alignment site with a Phylop conservation score of 4.5 and leading to the amino acid substitution I184F. This variant is predicted by Polyphen-2 to be probably damaging with a score of 1. Exome variant analysis in family 187 revealed a mutation at a site with a Phylop conservation score of 1.4, leading to the substitution I184M. The Polyphen score was also 1, with a high probability of being damaging. This variant has been previously described in a family with DCM, left ventricular non-compaction, atrial and ventricular septal defects and conduction system disease [9]. Investigation of the functional effect of this substitution revealed impaired DNA binding with lowered transcriptional activity.

Following identification of these potentially pathogenic mutations in the probands, extended family members underwent clinical evaluation and PCR-based genotyping. Seventeen members of family 186 and nine members of family 187 underwent a standardized interview and physical examination. 12-lead electrocardiogram and echocardiogram were performed where clinically indicated, or in case of a mutation being identified. Affected individuals were identified as those with a self-reported history of cardiac disease such as septal defect closure in infancy, conduction system or rhythm abnormalities on the electrocardiogram or evidence of septal defect or DCM during echocardiographic analysis.

### Availability of data and materials

Disease variants discovered in the present study will be uploaded to the Database of Genotypes and Phenotypes (dbGaP), Bethesda (MD): National Center for Biotechnology Information, National Library of Medicine. Available from: http://www.ncbi.nlm.nih.gov/sites/entrez?db=gap

## Confirmation sequencing

Sanger sequencing was used to confirm the presence of variants and to perform segregation analysis. Oligonucleotide primers flanking the putative variants were designed using genomic sequences from the UCSC Genome Browser (hg19 assembly). Standard conditions were used to perform PCR. DNA sequencing was performed using the ABI PRISM dye terminator method (Applied Biosystems, CA, USA).

## Segregation analysis

In family 186, the proband is individual IV.1. Her sons (V.1 and V.2) demonstrated evidence of cardiac disease. V.1 had a history of conduction system disease requiring pacemaker implant at the age of 15. At the screening visit a septal defect was apparent on echocardiography, with the atrial pacemaker lead crossing through it to the left atrium. V.2 had first degree heart block evident on 12-lead ECG. In addition, individual IV.4 had a cardiac septal defect repaired surgically in infancy. At the screening visit he was found to be in atrial fibrillation, with mildly impaired LV function on echo. All of these affected individuals were found to carry the I184F variant. Other individuals (IV.3, IV.5) harboring the mutation had normal cardiac investigations, despite being older than other clinically affected carriers, indicating incomplete penetrance. Individual IV.4 had two young children (not shown) who are also carriers without evidence of disease, possibly due to their young age.

Several affected individuals were unavailable for genetic testing. Specifically, the proband’s father, III.2 had a pacemaker implanted while in his fourth decade. His brother, III.3 also had a pacemaker. A paternal great-uncle, II.3, had a pacemaker and his daughter, III.4, suffered sudden death at the age of nineteen. Individual II.6 died suddenly at age 3. III.5 was diagnosed with DCM and VT. His daughter, IV.6 had an atrial septal defect and died suddenly at the age of 15. Her paternal uncle, III.6 had DCM and AF, and died suddenly aged 51.

In family 187, the proband is individual III.1. Her sister (III.2) was diagnosed with DCM just prior to enrollment. A first-degree cousin, III.4, had a history of DCM requiring transplantation. His daughter (IV.1) had a septal defect corrected surgically in infancy. These affected individuals were all carriers of the I184M variant. Other members of the pedigree had cardiac disease but did not carry the variant. Individual III.6 had a history of non-sustained polymorphic VT documented on 24 hour Holter monitor. This may have been related to extreme caffeine intake at the time of the recording. Her father who died suddenly at a young age (II.5) had documented coronary disease at post mortem examination which was listed as the cause of death. Individual III.9 has a ventricular septal defect, despite not carrying the mutation. As her parents are mutation negative and are both healthy, it is probable that this is a sporadic phenomenon.

Some affected individuals were unavailable for genetic testing; the proband’s father (II.1) had a cardiac device implanted. His death was non-cardiac. His brother, II.3 had a pacemaker implanted aged 33 and died suddenly. His sister, II.7 had complex congenital cardiac disease including tricuspid atresia, right ventricular hypoplasia, pulmonary stenosis and a ventricular septal defect requiring a bidirectional Glenn shunt. Individual II.10 had cyanosis and died suddenly in infancy. Another sibling (not shown) was stillborn. Individual III.3 died suddenly aged 22.

## Discussion

*NKX2.5* is a highly conserved homeobox transcription factor that has a crucial role in cardiac development and homeostasis, specifying critical elements such as troponin T, connexin 43, β-myosin heavy chain and myosin light chain-2 [10, 11]. The gene is located on chromosome 5q34, spanning 2 exons and encoding a 324 amino acid homeodomain-containing protein [12]. Mutations in this gene cause a phenotypically broad array of structural cardiac defects and arrhythmias with variable penetrance [13]. The most common of these include septal defects and conduction system disease, supported by a recent review of reported mutations [14–16]. While we observed these phenotypes among families 186 and 187, the index cases presented with dilated cardiomyopathy. This was also the most common diagnosis among the extended pedigrees. We also observed incomplete penetrance, the cause for which is poorly understood but may relate to maternal factors [17].

On the basis of the conservation score, expression profile, predicted effect on protein function and segregation with disease in the families, I184F-NKX2.5 and I184M-NKX2.5 represent compelling candidate variants for the broad spectrum of cardiac disease in families 186 and 187 respectively. Previously reported functional studies provide further evidence to support the role of variants in I184-NKX2.5 in congenital cardiac disease [9].

The management of families with complex inherited cardiac disease is particularly challenging. Clinical testing can only diagnose mutation carriers expressing a clear phenotype. As mutations in some genes-including *NKX2.5*-may exert their effect in a time-dependent manner, longitudinal screening of unaffected individuals may be required [18]. Presentation may also include sudden death, and in the absence of a genetic diagnosis it can be difficult to identify at-risk individuals. An informative genetic test may therefore facilitate targeted screening and spare mutation negative individuals from unnecessary follow up and investigation.

In terms of achieving a molecular diagnosis, the pleiotropic nature of the conditions affecting families 186 and 187 does not implicate a particular gene or set of genes. The overall yield of genetic testing for cardiomyopathies employing current panels is 50–60% in the best cases (such as arrhythmogenic right ventricular cardiomyopathy, or familial hypertrophic cardiomyopathy [19–21]). Often the yield is either significantly lower than 50% or may be unknown [22]. The yield for testing in channelopathies is similarly unimpressive, with the lone exception of long QT syndrome [22]. In addition, *NKX2.5* is not included in most commercially available diagnostic gene panels for either cardiomyopathy or channelopathy [23–25].

Relying on traditional diagnostic methods would therefore have failed to yield a genetic diagnosis in families 186 and 187. Next generation sequencing provides a rapid and unbiased approach of evaluating the exome, greatly expanding the scope of genes in which mutations can be identified. This allowed identification of *NKX2.5* variants in each of our probands. Identification of these variants had important diagnostic and management implications for the extended family. The discovery facilitated cascade screening and enabled us to both arrange appropriate surveillance for otherwise healthy mutation positive individuals, and conversely to reassure and discharge healthy mutation negative individuals from the clinic.

It is a remarkable coincidence that two families living so close to each other with such a rare inherited disease did not share the same mutation, refuting our initial suspicion that a founder mutation would be discovered. An additional coincidence is the genomic proximity of the variants found in each study family with distinct mutations affecting not only the same rarely-implicated gene but also the same residue.

## Conclusion

Next generation sequencing and *in silico* variant prioritization may allow for rapid identification of pathogenic and novel mutations in complex pedigrees with pleiotropic cardiac disease. I184F-NKX2.5 is a novel variant associated with congenital heart disease.

## Abbreviations

AF: Atrial Fibrillation
CHD: Congenital Heart Disease
DCM: Dilated Cardiomyopathy
DNA: Deoxyribonucleotide Acid
ECG: Electrocardiogram
LV: Left Ventricle
MGH: Massachusetts General Hospital
PCR: Polymerase Chain Reaction
UCSC – University of California: Santa Cruz
VT: Ventricular Tachycardia
